# Inhibitory Activity of* Ficus deltoidea* var.* trengganuensis* Aqueous Extract on Lipopolysaccharide-Induced TNF-*α* Production from Microglia

**DOI:** 10.1155/2017/2623163

**Published:** 2017-11-22

**Authors:** Huiling Wang, Sharmili Vidyadaran, Mohamad Aris Mohd Moklas, Mohamad Taufik Hidayat Baharuldin

**Affiliations:** ^1^Department of Basic Medicine, Chengde Medical University, Hebei, China; ^2^Department of Human Anatomy, Faculty of Medicine and Health Sciences, Universiti Putra Malaysia, Selangor, Malaysia; ^3^Department of Pathology, Faculty of Medicine and Health Sciences, Universiti Putra Malaysia, Selangor, Malaysia

## Abstract

**Objective:**

To explore the effect of* Ficus deltoidea* (FD) aqueous extracts on the release of tumor necrosis factor-*α* (TNF-*α*), the expression of CD40, and the morphology of microglial cells in lipopolysaccharide- (LPS-) activated BV2 cells.

**Methods:**

The cytotoxicity of FD extract was assessed by MTS solution. BV2 cells were divided into 5 experimental groups, intervened, respectively, by FD (4 mg/mL) and LPS + FD (0, 1, 2, and 4 mg/mL). Besides, a blank control group was set up without any intervention. TNF-*α* release was assessed by enzyme linked immunosorbent assay (ELISA). The expression of CD40 was examined by flow cytometry. Immunocytochemical staining was used to show the morphology of BV2 cells.

**Results:**

FD extract of different concentrations (1, 2, and 4 mg/mL) had no significant toxic effects on the BV2 cells. FD suppressed the activation of microglia in morphology and reduced TNF-*α* production and expression of CD40 induced by LPS.

**Conclusion:**

FD extract has a therapeutic potential against neuroinflammatory diseases.

## 1. Introduction

Neuronal damage in neurodegenerative diseases is associated with neuroinflammatory responses [1], whose hallmark is the activation of microglia [2]. Microglia originate in the extraembryonic yolk sac during embryonic development and almost evenly populate throughout the whole brain [3]. They act as the main and first form of active immune defense in CNS. Under resting conditions, microglia in a ramified state support the proper function of neurons, organize and preserve the neuronal network, and maintain homeostasis [4, 5]. When brain homeostasis is disturbed, such as in injury, infection, or obvious alterations of neuronal activity, microglia rapidly transform into an amoeboid morphology, acquire the ability to proliferate and migrate, and secrete inflammatory mediators [6].

TNF-*α*, one of the proinflammatory cytokines, plays an important role in the initiation and regulation of inflammatory responses. Studies support the notion that TNF-*α* has neurotoxic effects on neuronal death both* in vivo* [7] and* in vitro* [8]. In CNS, TNF-*α* produced by activated microglia can further activate microglia and astrocytes to enhance the inflammatory response by cascade amplification [9]. In addition, TNF-*α* can stimulate extensive glutamate release from microglia via the upregulation of glutamate synthesis and downregulation of glutamate uptake [10]. Therefore, the level of TNF-*α* is a key step in neurodegenerative diseases and inhibition of TNF-*α* production from microglia may be an effective strategy against the neuronal damage mediated by TNF-*α*.

*Ficus deltoidea* (FD), known as “Mas Cotek” in Malaysia, is used in traditional medicine to treat various kinds of ailments such as sores, wounds, pain, and rheumatism [11]. Many studies confirmed that FD possessed strong anti-inflammatory effects in some inflammatory models [12–14]. It was reported that FD leaves extract reduces serum levels of IL-1*β* and PGE_2_ in osteoarthritis rats [12]. Aqueous extracts of three varieties of* Ficus deltoidea* showed different anti-inflammatory activities against lipoxygenase, hyaluronidase, and 12-O-tetradecanoylphorbol 13-acetate- (TPA-) induced ear edema [14]. Therefore, it is likely that the anti-inflammatory effects of FD are common. The aim of this present study was to reveal the potential and possible mechanism through which FD extract suppresses the activation of LPS-treated BV2 cells.

## 2. Methods

### 2.1. *Ficus deltoidea*

Plant samples of FD var.* trengganuensis* were collected from a farm in Malacca. After being air-dried, the leaves were coarsely powdered and then extracted with boiling water for 1 h. The infusion was filtered and the filtrate was spray-dried to form a powder. A voucher specimen was deposited at Universiti Kebangsaan Malaysia herbarium. The voucher specimen number is UKMB 40354. The powder was dissolved and diluted to suitable concentrations with sterile water before usage.

### 2.2. BV2 Cell Culture

The murine microglial cell line BV2 was obtained from Assoc. Professor Dr. Thameem Dheen of the National University of Singapore. BV2 cells were cultured in Dulbecco's modified Eagle's medium (DMEM, Gibco, USA) containing 5% heat-inactivated fetal bovine serum (FBS) (iDNA, South America), 1% (v/v) penicillin and streptomycin, and 0.3% (v/v) insulin in fully humidified air of 5% carbon dioxide (CO_2_) at 37°C.

### 2.3. Cell Viability Assay

MTS solution (Promega, USA) was used to determine whether the FD concentrations used in the experiment caused any cytotoxicity in BV2 cells. This assay is based on the mitochondrial mediated reduction of a tetrazolium compound (MTS) by living cells to form a colored formazan product which is measured colorimetrically [15, 16]. Briefly, cells were seeded on a 96-well plate at the density of 5 × 10^4^ cells/well. After 24 h, the cells were incubated with FD (0, 1, 2, 4, and 8 mg/mL) for 24 h or 48 h. 20 *μ*L of MTS/PMS solution (Promega, USA) was added to each well. After 3 h incubation at 37°C in 5% CO_2_, the concentration of the MTS formazan product was measured at 490 nm (Dynex MRX II microplate reader). The average of absorbance values for triplicate wells was examined. The absorbance values of all wells were deducted with the values of DMED-treated control, which served as the background reading, and reported in percentage (%) as cell viability.

### 2.4. Immunocytochemical Staining of BV2 Cells

BV2 cells were seeded onto the culture slides treated with poly-L-lysine for 24 h before being preincubated with FD (4 mg/mL) for 24 h. Then, the cells were treated with 1 *μ*g/mL LPS or normal medium for 12 h. After being washed with PBS thrice for 5 min, the cells were fixed by 4% paraformaldehyde for 1 h at room temperature. 0.2% Triton-X was added to cultures and incubated at 4°C for 30 minutes. Subsequently, the cells were stained with Lectin from* Lycopersicon esculentum* (1 : 300). After being washed with PBS, the cells were counterstained with DAPI (1 : 1000, Invitrogen, USA; cat. number D1306) at 4°C for 20 min, washed with PBS, mounted onto microscope slides, and sealed.

### 2.5. CD40 Immunophenotyping

BV2 cells (1 × 10^5^ cells/mL) were pretreated with FD (0, 1, 2, and 4 mg/mL) for 24 h in 24-well plates before being incubated with 1 *μ*g/mL LPS for 24 h. Then, the cells were harvested and resuspended in PBS. CD40-FITC antibody (1 : 100; BD Pharmingen, San Diego, USA) and Fixable Viability Dye eFluor™ 780 (1 : 1000; eBioscience, San Diego, USA) were used for immunofluorescence staining. Cells were analyzed by a FACS Fortessa Cytometer (BD Biosciences, San Jose, CA, USA). The FACSDiva software was used to analyze the data.

### 2.6. ELISA for TNF-*α*

ELISA kit (BD Sciences, San Jose, CA, USA) was used to assess the inhibitory effect of FD on TNF-*α* production. BV2 cells (5 × 10^4^ cells/mL) were pretreated with FD (0, 1, 2, and 4 mg/mL) for 24 h in 96-well plates before being incubated with 1 *μ*g/mL LPS for 12 h. According to the manufacturers' protocol, 100 *μ*L of supernatants was collected in each well of the ELISA plate. The cell absorbance was then read by a microplate reader at 450 nm. The concentration in each sample was calculated according to the standards provided with the kit.

### 2.7. Statistical Analysis

Data were presented as mean ± SEM. All data were analyzed by one-way ANOVA followed by Tukey's post hoc test with GraphPad Prism Software version 5.0. The group means were considered significantly different at the level of *p* < 0.05.

## 3. Discussion

Under some pathological conditions, such as infection, trauma, and ischemia, microglia can be activated. Activated microglia act as the first defense in the brain, regulating the expression of some immune-related molecules and releasing cytokines and chemokines. At the same time, they can also engulf invasive pathogens, harmful substances, and debris of dead neurons, so as to play a protective role in neurons. On the other hand, the sustained activation of microglia makes them secrete a series of toxic substances and proinflammatory factors, such as reactive oxygen species (ROS), interleukin-1*β* (IL-1*β*), TNF-*α*, and NO [1, 17]. These substances cause the amplification of inflammatory reaction in the CNS, with higher release of toxic products, leading to neuronal degeneration and necrosis in the corresponding brain, followed by the emergence of the corresponding disease. Therefore, inhibition of activated microglia may be the key to inhibit neuroinflammatory response. In the present study, FD reduced the release of TNF-*α* from LPS-activated BV2 cells by the inhibition of CD40 signaling pathway.

In normal medium or 4 mg/mL FD alone, most of the BV2 cells are composed of a small cellular body and some bipolar projections. In this state, the main functions of microglia are to search for immune threats and to maintain homeostasis in the CNS. Resting microglia are extremely sensitive to even small pathological changes and undergo various structural and functional changes based on their role and location in response to injury or threat [18]. LPS is a bacterial cell wall component which triggers microglia activation via Toll-like receptor 4 [19]. After LPS treatment, BV2 cells became round, retracting the branches. Some of them showed an increased volume and an amoeboid morphology. However, preincubation with FD attenuated this morphological change, indicating its inhibition in LPS-activated BV2 cells.

CD40, a member of the TNF receptor superfamily (TNFR), is a 50 kDa type I phosphoprotein [20]. CD40 expression on microglia is an important component of the neuroinflammatory response in the CNS. Stimulation of rat CD40^+^ microglia with LPS leads to higher neurotoxic iNOS and TNF-*α* mRNA expression than stimulation of CD40^−^ microglia [21]. LPS and IFN-*γ* dramatically induced CD40 expression in cultured microglia [22, 23]. Activated CD4^+^ T-cells, astrocytes, macrophages, vascular endothelial cells, and smooth muscle cells have the capacity to express the ligand for CD40. Ligation of CD40 activates many signaling pathways including NF-*κ*B, MAPK, and TRAF proteins and P13K and JAK/STAT pathways which lead to alterations in gene expression and function [24]. The immune-activation pathway of CD40-CD40L is closely related to both the host response against infection and the development of autoimmune diseases [25, 26]. The interaction between CD40 and CD40L promotes the production of various neurotoxins from microglia including NO, TNF-*α*, IL-12, MCP-1, MMP-9, and IP-10, as well as some unidentified ones. So, a decrease in the expression of CD40 may be beneficial to extenuate neuroinflammation and protect neurons within the CNS [27]. In the present study, the expression of CD40 in resting BV2 cells was at a low level, which was in accordance with previous literatures [22]. However, the expression of CD40 increased in FD-alone group. Perhaps FD existed in the supernatant like some foreign matters to the microglia, so that microglia expressed more CD40 and were activated to engulf and eliminate them. Microglia stimulated by LPS showed a higher number of cells with basal expression of CD40 compared to that in control and FD-alone group, which was reduced by preincubation with 4 mg/mL FD. The two-step activation process of microglia during autoimmune inflammation in CNS required CD40 expression on microglial cells [28]. The fully activated microglia present antigens and stimulate T-cells, leading to the exacerbation of disease. Therefore, FD may prevent antigen-presenting functions of microglia by reducing their CD40 expression. Inhibition of microglial activation by suppression of CD40 expression to attenuate inflammation within the CNS may be a beneficial strategy for neuroinflammatory diseases.

TNF-*α*, one of the proinflammatory cytokines involved in systemic inflammation, is mainly produced by activated macrophages. In the CNS, the primary source of TNF-*α* is activated microglia followed by activated astrocytes. The main role of TNF is to regulate immune cells. TNF can cause fever, inflammation, apoptosis, and cachexia and inhibit viral replication and tumorigenesis. TNF-*α* plays a central role in the initiation and regulation of inflammatory responses. LPS-activated microglia can secrete a lot of TNF-*α*, which can activate microglia and astrocytes to increase the inflammatory response by cascade amplification [29]. TNF-*α* and glutamate acted synergistically to induce the expression of various inflammatory-related factors, neurotoxic effects, and neuronal cell death [30, 31]. In the present study, microglia in normal medium or incubated with FD hardly produced TNF-*α*. After LPS stimulation, BV2 cells release more than 600 pg/mL TNF-*α*, which reduced by different doses of FD. This also proved that FD inhibited the activation of microglia induced by LPS.

FD has been used as a traditional medicine in Malaysia to treat various kinds of ailments such as sores, wounds, pain, and diabetes. Acute and chronic inflammatory models were used to evaluate the anti-inflammatory activity of FD aqueous extract [13]. A study indicated that FD leaf extract had significant anti-inflammatory properties [14]. So far, it has not been reported whether FD can inhibit the activation of microglia induced by LPS. In the present study, FD significantly ameliorated TNF-*α* release and CD40 expression induced by LPS in a dose-dependent manner. Data from morphology also demonstrated that FD pretreatment inhibited microglial activation. Cell viability assay showed that FD (1, 2, and 4 mg/mL) had no significant toxic effects on BV2 cells, which excluded the possibility that the inhibitory effect was due to a decrease of cell viability. This suggested that anti-inflammatory effects of FD might be common. The anti-inflammatory effects of FD might be associated with the inhibition of activated microglia by CD40 pathway. The active components in FD extract further identification and purification.

## 4. Results

### 4.1. Effects of FD on the Viability of BV2 Cells

**Figure 1:**
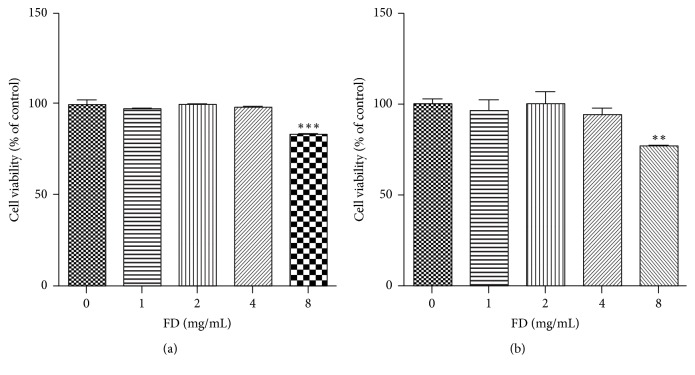
Effect of FD on BV2 cell viability. BV2 cells were treated with different concentrations of FD for 24 h (a) and 48 h (b) and cell viability was detected by MTS conversion. FD (1, 2, and 4 mg/mL) had no significant toxic effects on BV2 cells. The data are expressed as mean ± SEM of three independent experiments. ^*∗∗*^*p* < 0.01 and ^*∗∗∗*^*p* < 0.001, compared to normal medium (0 mg/mL FD).

To examine the toxicity of FD, BV2 cells were incubated with or without FD at the specified concentrations for 24 h and 48 h. The cell viability was detected by MTS. As shown in Figure 1, FD of different concentrations (1, 2, and 4 mg/mL) had no significant toxic effects on the cells (*p* > 0.05). The cell viability is approximately 100%. But FD of 8 mg/mL reduced it. Therefore, we used these three concentrations of FD in subsequent experiments.

### 4.2. Effects of FD on LPS-Induced Microglial Activation

**Figure 2:**
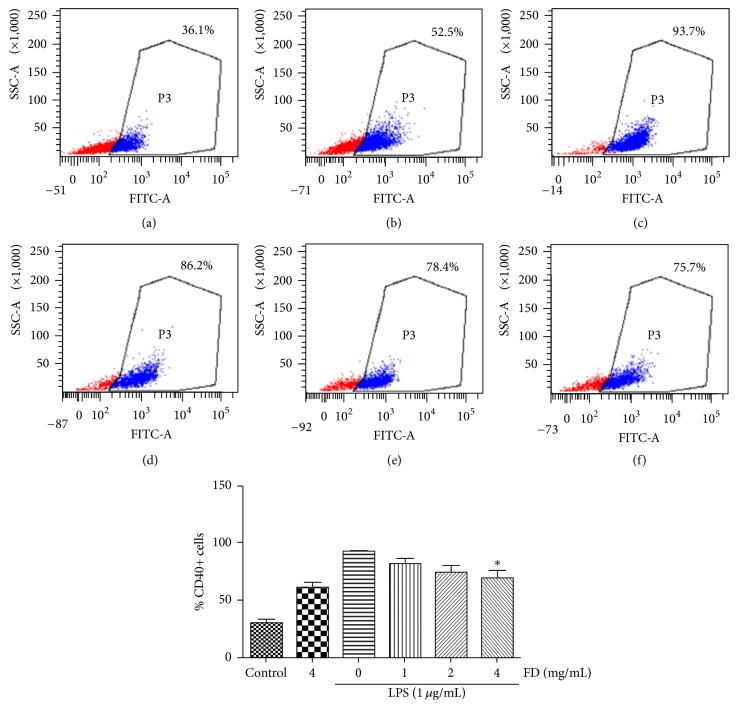
Effects of FD on CD40 expression in LPS-treated BV2 cells. BV2 cells were preincubated in a medium containing 0, 1, 2, and 4 mg/mL FD for 24 h and then activated by 1 *μ*g/mL LPS for 24 h. The CD40 expression on BV2 cells was analyzed by flow cytometry. CD40^+^ BV2 cells in 4 mg/mL FD were decreased significantly compared to LPS treatment alone. The results shown were from one representative experiment of three independent experiments performed. (a) Normal medium; (b) FD (4 mg/mL); (c) LPS (1 *μ*g/mL); (d) LPS + FD (1 mg/mL); (e) LPS + FD (2 mg/mL); (f) LPS + FD (4 mg/mL).

For morphological analysis, we labeled BV2 cells with Lectin from* Lycopersicon esculentum* and DAPI to show the size and shape of the cells.  Figure 2 shows typical composite images of labeled BV2 imaged at 20x magnification. As shown in Figure 2, BV2 cells maintained the resting state in normal medium and 4 mg/mL FD alone. Most of them exhibited round cytoplasm with some bipolar projections. However, in the LPS group, the extent of amoeboid morphology seemed to be increased with the cytoplasmic area appearing minimal. But preincubation with 4 mg/mL FD attenuated LPS-induced morphological changes of BV2 cells. The number of BV2 cells with bipolar projections increased obviously compared with the LPS group.

### 4.3. Effects of FD on CD40 Expression

**Figure 3:**
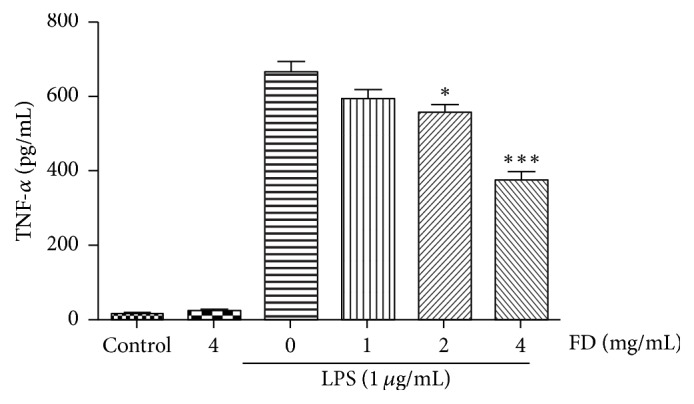
Effect of FD on TNF-*α* release in LPS-treated BV2 cells. FD (2 and 4 mg/mL) decreased TNF-*α* release induced by LPS. The data are expressed as mean ± SEM of three independent experiments (^*∗*^*p* < 0.05 and ^*∗∗∗*^*p* < 0.001, compared to LPS-alone group).

CD40 is a measure of the activated microglia. LPS treatment induced microglia to release inflammatory mediators and express major histocompatibility complex (MHC) class II receptors and CD40 to facilitate antigen presentation to T-lymphocytes [19]. We assessed the level of the costimulatory molecule CD40, one of the members of the TNF receptor superfamily, with flow cytometry. As shown in Figure 3, the number of CD40^+^ BV2 cells in the control group was at a low level, approximately 30%. However, the number of CD40^+^ BV2 cells increased by about 63% upon exposure to LPS. Almost all of the BV2 cells expressed CD40 (93.0 ± 4.0%) after LPS treatment for 24 h. However, CD40^+^ BV2 cells in FD groups (1, 2, and 4 mg/mL) decreased by 10.4%, 18.2%, and 23.4%, respectively, compared with LPS treatment alone, indicating the inhibition of activation of BV2 cells.

### 4.4. Effects of FD on TNF-*α* Release in LPS-Treated BV2 Cells

**Figure 4:**
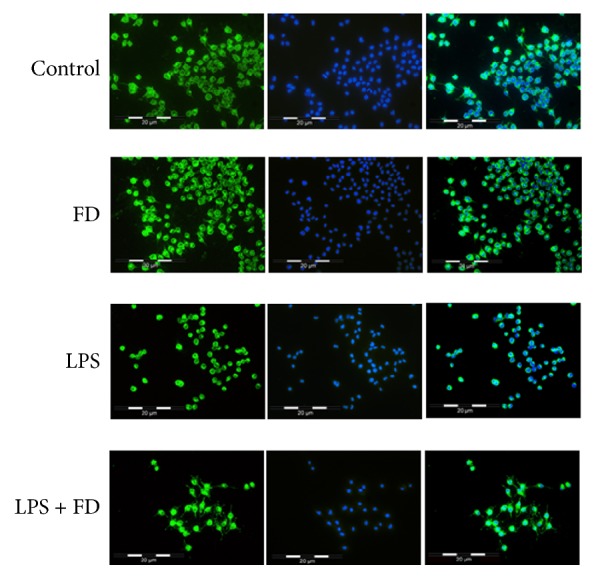
Composite immunofluorescent images of stained BV2 cells. Cell bodies are shown in green, and the DAPI stained nuclei are shown in blue. Pretreatment with 4 mg/mL FD attenuated the morphological changes of BV2 cells induced by LPS. Control: untreated BV2 cells. FD: BV2 cells treated with FD (4 mg/mL) for 24 h. LPS: BV2 cells treated with LPS (1 *μ*g/mL) for 12 h. LPS + FD: BV2 cells treated with LPS (1 *μ*g/mL) for 12 h after being preincubated with FD (4 mg/mL) for 24 h. Images were captured at 20x objective magnification.

We examined the release of an important inflammatory cytokine, TNF-*α*. As shown in Figure 4, the levels of TNF-*α* were less than 31 pg/mL in the supernatant of BV2 cells treated with normal medium or FD (4 mg/mL) alone. LPS treatment increased TNF-*α* release to about 22-fold higher than that in the control group. FD of 1 mg/mL decreased the release of TNF-*α*, but this was not statistically significant. LPS-induced release of TNF-*α* was significantly ameliorated by FD pretreatment (2 and 4 mg/mL). In particular, FD of 4 mg/mL decreased LPS-induced TNF-*α* release to nearly 332 pg/mL.

## 5. Conclusions

FD extract can inhibit LPS-induced activation of microglia by reducing TNF-*α* release and CD40 expression.

## References

[1] Liu B., Hong J.-S. (2003). Role of microglia in inflammation-mediated neurodegenerative diseases: mechanisms and strategies for therapeutic intervention. *The Journal of Pharmacology and Experimental Therapeutics*.

[2] González-Scarano F., Baltuch G. (1999). Microglia as mediators of inflammatory and degenerative diseases. *Annual Review of Neuroscience*.

[3] Ginhoux F., Greter M., Leboeuf M. (2010). Fate mapping analysis reveals that adult microglia derive from primitive macrophages. *Science*.

[4] Paolicelli R. C., Bolasco G., Pagani F. (2011). Synaptic pruning by microglia is necessary for normal brain development. *Science*.

[5] Schafer D. P., Lehrman E. K., Kautzman A. G. (2012). Microglia sculpt postnatal neural circuits in an activity and complement-dependent manner. *Neuron*.

[6] Wang H., Liu Y., Zhang J. (2016). 15-O-Acetyl-3-O-benzoylcharaciol and helioscopinolide A, two diterpenes isolated from Euphorbia helioscopia suppress microglia activation. *Neuroscience Letters*.

[7] Haile W. B., Wu J., Echeverry R., Wu F., An J., Yepes M. (2012). Tissue-type plasminogen activator has a neuroprotective effect in the ischemic brain mediated by neuronal TNF-*α*. *Journal of Cerebral Blood Flow & Metabolism*.

[8] Morini R., Ghirardini E., Butti E., Verderio C., Martino G., Matteoli M. (2015). Subventricular zone neural progenitors reverse TNF-alpha effects in cortical neurons. *Stem Cell Research & Therapy*.

[9] Block M. L., Hong J. S. (2005). Microglia and inflammation-mediated neurodegeneration: multiple triggers with a common mechanism. *Progress in Neurobiology*.

[10] Chen C.-J., Ou Y.-C., Chang C.-Y. (2012). Glutamate released by Japanese encephalitis virus-infected microglia involves TNF-*α* signaling and contributes to neuronal death. *Glia*.

[11] Hassan W. E. (2006). *Healing Herbs of Malaysia*.

[12] Ahmad Tantowi N. C., Mohamed S., Hussin P. (2016). Effect of ficus deltoidea, a medicinal plant, on cartilage protection in cartilage explant and postmenopausal rat models of osteoarthritis. *Osteoarthritis and Cartilage*.

[13] Zakaria Z. A., Hussain M. K., Mohamad A. S., Abdullah F. C., Sulaiman M. R. (2012). Anti-inflammatory activity of the aqueous extract of ficus deltoidea. *Biological Research for Nursing*.

[14] Zunoliza A., Khalid H., Zhari I., Rasadah M. A. (2009). Anti-inflammatory Activity of Standardised Extracts of Leaves of Three Varieties of Ficus deltoidea. *International Journal of Pharmaceutical and Clinical Research*.

[15] Cory A. H., Owen T. C., Barltrop J. A., Cory J. G. (1991). Use of an aqueous soluble tetrazolium/formazan assay for cell growth assays in culture. *Cancer Communications*.

[16] Malich G., Markovic B., Winder C. (1997). The sensitivity and specificity of the MTS tetrazolium assay for detecting the *in vitro* cytotoxicity of 20 chemicals using human cell lines. *Toxicology*.

[17] Rezaie P., Trillo-Pazos G., Everall I. P., Male D. K. (2002). Expression of *β*-chemokines and chemokine receptors in human fetal astrocyte and microglial co-cultures: Potential role of chemokines in the developing CNS. *Glia*.

[18] Aloisi F. (2001). Immune function of microglia. *Glia*.

[19] Olson J. K., Miller S. D. (2004). Microglia initiate central nervous system innate and adaptive immune responses through multiple TLRs. *The Journal of Immunology*.

[20] Schönbeck U., Libby P. (2001). The CD40/CD154 receptor/ligand dyad. *Cellular and Molecular Life Sciences*.

[21] Kawahara K., Yoshida A., Koga K. (2009). Marked induction of inducible nitric oxide synthase and tumor necrosis factor-*α* in rat CD40+ microglia by comparison to CD40- microglia. *Journal of Neuroimmunology*.

[22] Qin H., Wilson C. A., Lee S. J., Zhao X., Benveniste E. N. (2005). LPS induces CD40 gene expression through the activation of NF-*κ*B and STAT-1*α* in macrophages and microglia. *Blood*.

[23] Qin H., Wilson C. A., Lee S. J., Benveniste E. N. (2006). IFN-beta-induced SOCS-1 negatively regulates CD40 gene expression in macrophages and microglia. *The FASEB Journal: Official Publication of the Federation of American Societies for Experimental Biology*.

[24] Van Kooten G., Banchereau J. (2000). CD40-CD40 ligand. *Journal of Leukocyte Biology*.

[25] Munroe M. E. (2009). Functional roles for T cell CD40 in infection and autoimmune disease: The role of CD40 in lymphocyte homeostasis. *Seminars in Immunology*.

[26] Peters A. L., Stunz L. L., Bishop G. A. (2009). CD40 and autoimmunity: the dark side of a great activator. *Seminars in Immunology*.

[27] Benveniste E. N., Nguyen V. T., Wesemann D. R. (2004). Molecular regulation of CD40 gene expression in macrophages and microglia. *Brain, Behavior, and Immunity*.

[28] Ponomarev E. D., Shriver L. P., Dittel B. N. (2006). CD40 expression by microglial cells is required for their completion of a two-step activation process during central nervous system autoimmune inflammation. *The Journal of Immunology*.

[29] Rubio-Perez J. M., Morillas-Ruiz J. M. (2012). A review: inflammatory process in Alzheimer's disease, role of cytokines. *The Scientific World Journal*.

[30] Zou J. Y., Crews F. T. (2005). TNF*α* potentiates glutamate neurotoxicity by inhibiting glutamate uptake in organotypic brain slice cultures: neuroprotection by NF*κ*B inhibition. *Brain Research*.

[31] Angelopoulos P., Agouridaki H., Vaiopoulos H. (2008). Cytokines in Alzheimer's disease and vascular dementia. *International Journal of Neuroscience*.

